# The epidemiological patterns of non-Hodgkin lymphoma: global estimates of disease burden, risk factors, and temporal trends

**DOI:** 10.3389/fonc.2023.1059914

**Published:** 2023-06-02

**Authors:** Yurou Chu, Yingyue Liu, Xiaosheng Fang, Yujie Jiang, Mei Ding, Xueling Ge, Dai Yuan, Kang Lu, Peipei Li, Ying Li, Hongzhi Xu, Juan Fan, Xiangxiang Zhou, Xin Wang

**Affiliations:** ^1^ Department of Hematology, Shandong Provincial Hospital, Shandong University, Jinan, Shandong, China; ^2^ Department of Hematology, Shandong Provincial Hospital Affiliated to Shandong First Medical University, Jinan, Shandong, China; ^3^ Shandong Provincial Engineering Research Center of Lymphoma, Jinan, Shandong, China; ^4^ Branch of National Clinical Research Center for Hematologic Diseases, Jinan, Shandong, China; ^5^ National Clinical Research Center for Hematologic Diseases, the First Affiliated Hospital of Soochow University, Suzhou, China

**Keywords:** non-Hodgkin lymphoma, global disease burden, epidemiology, incidence, mortality, lifestyle

## Abstract

**Background:**

The incidence of non-Hodgkin’s lymphoma (NHL) has increased steadily over the past few decades. Elucidating its global burden will facilitate more effective disease management and improve patient outcomes. We explored the disease burden, risk factors, and trends in incidence and mortality in NHL globally.

**Methods:**

The up-to-date data on age-standardized incidence and mortality rates of NHL were retrieved from the GLOBOCAN 2020, CI5 volumes I-XI, WHO mortality database, and Global Burden of Disease (GBD) 2019, focusing on geographic disparities worldwide. We reported incidence and mortality by sex and age, along with corresponding age-standardized rates (ASRs), the average annual percentage change (AAPC), and future burden estimates to 2040.

**Results:**

In 2020, there were an estimated 545,000 new cases and 260,000 deaths of NHL globally. In addition, NHL resulted in 8,650,352 age-standardized DALYs in 2019 worldwide. The age-specific incidence rates varied drastically across world areas, at least 10-fold in both sexes, with the most pronounced increase trend found in Australia and New Zealand. By contrast, North African countries faced a more significant mortality burden (ASR, 3.7 per 100,000) than highly developed countries. In the past decades, the pace of increase in incidence and mortality accelerated, with the highest AAPC of 4.9 (95%CI: 3.6-6.2) and 6.8 (95%CI: 4.3-9.2) in the elderly population, respectively. Considering risk factors, obesity was positively correlated with age-standardized incidence rates (P< 0.001). And North America was the high-risk region for DALYs due to the high body mass index in 2019. Regarding demographic change, NHL incident cases are projected to rise to approximately 778,000 by 2040.

**Conclusion:**

In this pooled analysis, we provided evidence for the growing incidence trends in NHL, particularly among women, older adults, obese populations, and HIV-infected people. And the marked increase in the older population is still a public health issue that requires more attention. Future efforts should be directed at cultivating health awareness and formulating effective and locally tailored cancer prevention strategies, especially in most developing countries.

## 1 Introduction

Non-Hodgkin lymphoma (NHL) comprises a diverse and heterogeneous group of cancers arising from lymphoid tissue with unresolved etiologies (1). The global burden of the NHL is substantial. Globally, the incidence of NHL has increased rapidly in the past decades. NHL ranked as the 11th most commonly diagnosed cancer, with nearly 545,000 new cases, and the 11th leading cause of cancer-related death in 2020, with an estimated 260,000 deaths (2). With the advent of novel targeted therapies, prognosis in NHL has significantly improved over the past few decades, with survival rates exceeding 80% in high-income countries (3). However, there remain marked variations in survival rates internationally, as five-year age-standardized net survival in Central and South America is below 50% (3).

Although the etiology of NHL is largely unknown, risk factors for NHL include pre-existing immune diseases, medications, infections, unhealthy lifestyles, ethnicity, genetics, heredity, and certain occupations (4–9). Some risk factors are considered preventable. For example, excess body weight in young adulthood has a measurable impact on the risk of NHL in the United States (10). According to this association, up to 11% of NHL cases may be prevented by avoiding obesity in adults.

NHL in the elderly also represents a substantial disease burden. Peak incidence occurs at age 75 or older, contributing significantly to overall incidence (11). Meanwhile, the size of elderly populations at risk of NHL is projected to increase in the coming decades, according to the aging and growth of the people. Hence, understanding and mitigating the global burden of NHL in an aging population is also an essential public health issue with wide-ranging economic implications, including medical care and in-home assistance.

Although several regional and national studies have been conducted on NHL morbidity and mortality, studies that have comprehensively assessed the burden and risk factors of NHL on a global scale are limited. Overall, we conducted this study to analyze geographic region-based incidence and mortality estimates by assessing age-standardized rates (ASRs) from GLOBOCAN 2020. We analyzed temporal trends in morbidity and mortality by assessing average annual percent changes (AAPCs) obtained from CI5 volumes I-XI and WHO mortality database to gain a comprehensive understanding of the global burden of NHL. We also examined the associated risk factors and disability-adjusted life years (DALYs), and provided global demographic projections for 2040.

## 2 Methods

### 2.1 Data collection

A population-based study was conducted to estimate global trends and the current and future burdens of NHL based on data from the GLOBOCAN 2020 database of the Global Cancer Observatory (GCO). Available data covering cancer incidence, mortality, and prevalence in 185 countries or regions were described on the GCO website (12). We profiled the burden in terms of the human development index (HDI), a three-dimensional index focusing on measuring the average achievement in crucial issues of human development: life expectancy, education, and gross national income (13).

We estimated associations between the age-adjusted prevalence of health-related risk factors and the global incidence and mortality data of NHL mostly in 2016 for each country from the WHO Global Health Observatory (GHO) database (14). When comparing the influence of risk factors on NHL, the prevalence data of risk factors were retrieved from people older than 18 years old, and the ASRs of incidence and mortality. A detailed definition of risk factors can be found in the GHO database or **Supplementary Table 1**. We investigated the correlation between HIV prevalence and the age standardized incidence rates (ASIRs) of NHL in people aged 15 to 49 in 93 counties (mainly in Africa) for the year 2020 from the website of AIDSinfo (13).

For the long-term trend analysis of NHL, data on cancer incidence and the corresponding populations at risk in 42 countries were collected from volumes VII-XI of the Five Continents Time Trends (CI5plus) database of the International Agency for Research on Cancer (IARC) covering the period from 1998 to 2012 (15). Annual mortality data were extracted from the WHO mortality database for the corresponding study period from 1998 to 2019 (16). The number of cases and deaths from NHL was extracted by sex and 18 age groups (0-4, 5-9,…, 80-84, 85 and over). Data from the Global Burden of Disease (GBD) 2019 study was used to estimate DALYs from 1990 to 2019 (17). We retrieved corresponding data of NHL from the cancer databases based on the codes (ICD-10: C82-86, C96). For the countries included in the study, the periods and countries analyzed were provided in **Supplementary Table 2**. To ensure comparisons between populations that differed concerning age structure, we tabulated ASRs per 100,000 population by country, sex, and age based on the Segi-Doll world standard population (18).

We also provided a simple estimate of the future number of NHL cases and deaths worldwide for 2040 based on demographic projections and the current global-level incidence and mortality rates of NHL for 2020. The predicted number of new cases or deaths was computed by multiplying the world’s age-specific incidence or mortality rates for 2020 by the corresponding projected world population estimate. The prediction assumed that national rates remain constant over the next two decades, so changes in the number of cases or deaths are based solely on the growth and aging of the population.

### 2.2 Statistical analysis

Data management and analyses were performed using R (version 4.1.2) software and Joinpoint Regression Program (version 4.3.1). Linear regression and locally weighted regression were used to assess the association between ASRs and HDI in age-specific groups of NHLs, as well as the correlation between lifestyle risk factors and incidence or mortality. For cases with unknown ages at diagnosis, they were not included in the analysis. The case fatality rate was defined as the age standardized mortality rate (ASMR) divided by the ASIR. The ASR (per 100,000 people) was calculated by multiplying the age-specific rate by the population of the same age subgroup of the selected reference standard population and dividing it by the sum of the standard population weights (19). A general formula for the ASR measure can be expressed as:


$$ASR=\frac{\sum_{i=1}^{A}a_{i}w_{i}}{\sum_{i=1}^{A}w_{i}}\times 100,000$$

(a*_i_*, where *i* denotes the *i*th age class and the number of persons (or weight) (w*_i_*) in the same age subgroup *i* of the selected reference standard population.)

We reported trends of NHL expressed as AAPCs with accompanying 95% confidence intervals (CIs) (20). The AAPC described disease occurrence in a population using a weighted average of the annual percent change. The AAPC for each interval was calculated as a weighted average of the slopes of the underlying Joinpoint linear regression lines. The final step of the calculation transformed the weighted average of slope coefficients to an annual percent change (21). The ASR was considered to increase if the AAPC estimate and the lower bound of its 95% CI were both > 0. We calculated DALYs as the sum of years of life lost due to premature mortality and years lived with disability (22). When we encountered missing data or zero values in the trend analysis, the corresponding countries were eliminated since the Joinpoint regression cannot be performed in this case. The two-sided P< 0.05 was regarded as statistically significant.

## 3 Results

### 3.1 Global estimates of NHL incidence and mortality rates in 2020 and comparison by HDI

**Figure 1:**
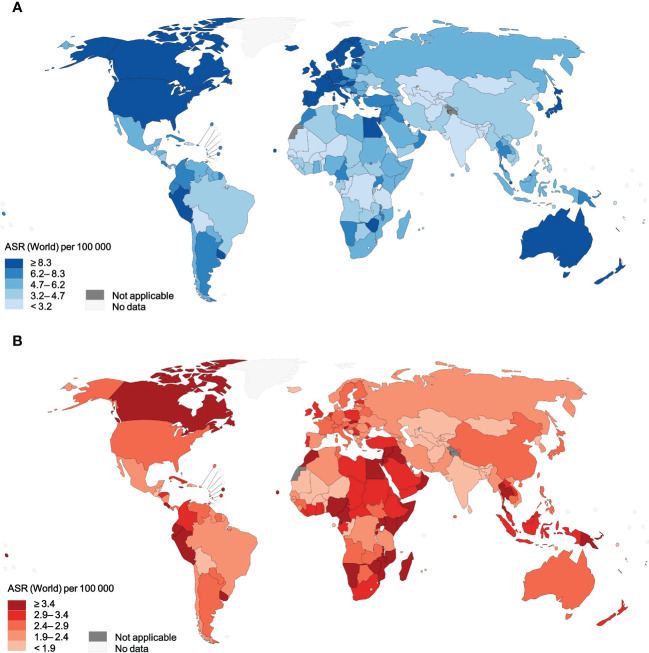
Age-standardized incidence rates and mortality rates of NHL for 185 countries or territories in 2020, for both sexes, all ages. **(A)** incidence. **(B)** mortality. NHL, non-Hodgkin lymphoma.

Overall, there were approximately 545 thousand new cases of NHL and 260 thousand related deaths worldwide in 2020. The crude rate and ASIR of NHL were 7.0 and 5.8 per 100,000 population on a global scale (**Table 1**). Age-specific incidence rates of NHL varied up to fifteen-fold, with a range of ASIRs from 13.3 (Israel) to 0.86 (Bhutan) for individual countries (**Figure 1**). A significant burden of NHL was recognized in high-income countries as the highest incidence rates were in Australia and New Zealand (ASR, 12.5 per 100,000 people), Northern America (ASR, 12.0 per 100,000 people), and Northern Europe (ASR, 11.4 per 100,000 people). The global crude rate and ASMR were 3.3 and 2.6 per 100,000 people in 2020, respectively. The distribution of mortality rates was considerably different from morbidity rates globally. Zimbabwe had the highest mortality rate (ASR, 6.5 per 100,000 people), while Albania had the lowest mortality rate (ASR, 0.59 per 100,000 people) (**Figure 1**). Regarding different continents or regions, higher mortality rates were seen in Melanesia (ASR, 4.0 per 100,000 people), North Africa (ASR, 3.7 per 100,000 people), and Micronesia (ASR, 3.6 per 100,000 people) (**Table 1**).

**Table 1:** Estimated number of cases, ASIR, ASMR, and case-fatality percentage for NHL in 2020.

Continents/Regions	Incidence					Mortality				Case fatality rate
	Cases	Proportion (%)	Crude rate	ASIR	Deaths		Proportion (%)	Crude rate	ASMR	
**World**	544352	100.0	7.0	5.8	259793		100.0	3.3	2.6	44.8%
Very high HDI	268797	49.4	17.2	9.3	101841		39.2	6.5	2.7	29.0%
High HDI	178556	32.8	6.1	4.8	99307		38.2	3.4	2.6	54.2%
Medium HDI	66556	12.2	2.9	3.1	38834		14.9	1.7	1.8	58.1%
Low HDI	30153	5.5	3.0	4.5	19687		7.6	2.0	3.2	71.1%
**Africa**	50516	9.3	3.8	5.2	30960		11.9	2.3	3.5	67.3%
Eastern Africa	15507	2.8	3.5	5.0	10071		3.9	2.3	3.5	70.0%
Northern Africa	14268	2.6	5.8	6.5	7969		3.1	3.2	3.7	56.9%
Western Africa	11974	2.2	3.0	4.5	7777		3.0	1.9	3.2	71.1%
Middle Africa	4988	0.9	2.8	3.6	3174		1.2	1.8	2.6	72.2%
Southern Africa	3779	0.7	5.6	5.8	1969		0.8	2.9	3.2	55.2%
**Asia**	241270	44.3	5.2	4.4	133407		51.4	2.9	2.4	54.5%
Eastern Asia	135359	24.9	8.1	5.0	73726		28.4	4.4	2.5	50.0%
South-Central Asia	52813	9.7	2.6	2.7	30372		11.7	1.5	1.6	59.3%
South-Eastern Asia	37027	6.8	5.5	5.2	21085		8.1	3.2	2.9	55.8%
Western Asia	16071	3.0	5.8	6.3	8224		3.2	3.0	3.3	52.4%
**Europe**	122979	22.6	16.4	8.4	49684		19.1	6.6	2.6	31.0%
Western Europe	43131	7.9	22.0	10.2	17423		6.7	8.9	2.8	27.5%
Southern Europe	28769	5.3	18.8	9.3	11284		4.3	7.4	2.5	26.9%
Central and Eastern Europe	26590	4.9	9.1	5.5	12669		4.9	4.3	2.3	41.8%
Northern Europe	24489	4.5	23.0	11.4	8308		3.2	7.8	2.8	24.6%
**Northern America**	82185	15.1	22.3	12.0	24179		9.3	6.6	2.7	22.5%
**Latin America and the Caribbean**	39886	7.3	6.1	5.2	19153		7.4	2.9	2.4	46.2%
South America	28110	5.2	6.5	5.4	13422		5.2	3.1	2.4	44.4%
Central America	8908	1.6	5.0	4.9	4182		1.6	2.3	2.2	44.9%
Caribbean	2868	0.5	6.6	5.3	1549		0.6	3.6	2.7	50.9%
**Oceania**	7516	1.4	17.6	11.7	2410		0.9	5.6	3.1	26.5%
Australia and New Zealand	6901	1.3	22.8	12.5	2061		0.8	6.8	2.8	22.4%
Melanesia	537	0.1	4.8	6.3	306		0.1	2.8	4.0	63.5%
Polynesia	50	0.0	7.3	6.9	23		0.0	3.4	3.2	46.4%
Micronesia	28	0.0	5.1	5.0	20		0.0	3.6	3.6	72.0%

ASIR, age-standardized incidence rate; ASMR, age-standardized mortality rate; NHL, non-Hodgkin lymphoma.

**Figure 2:**
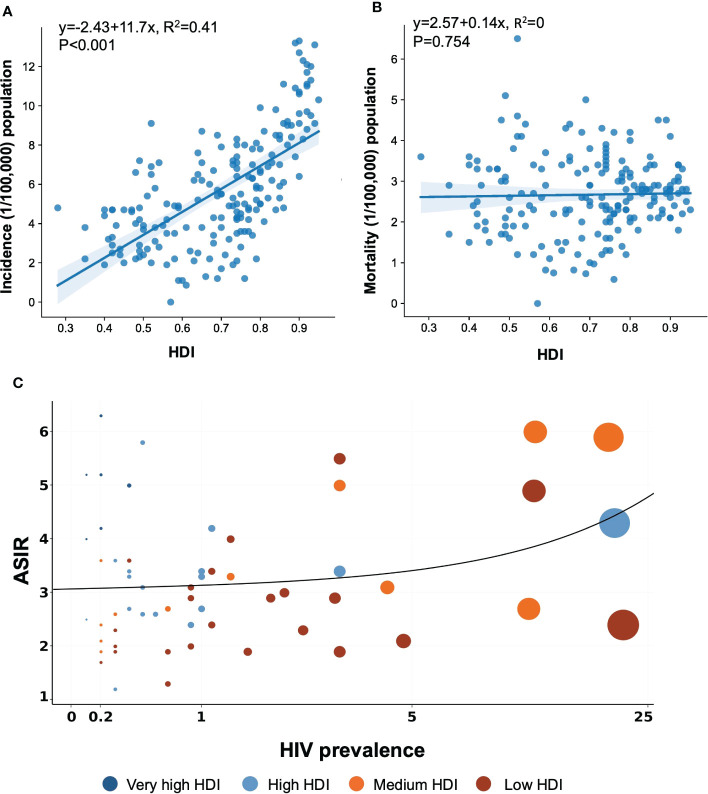
Association between HIV prevalence and HDI and age-standardized incidence and mortality rates of NHL in 2020. The data was obtained from the GLOBOCAN database and WHO Global Health Observatory, United Nations Development Programme in 2020. **(A)** Association between incidence and HDI. **(B)** Association between mortality and HDI. **(C)** Association between HIV prevalence and age-standardized incidence. NHL, non-Hodgkin lymphoma; HDI, Human Development Index.

**Table 1** shows the estimated burden of NHL by HDI category. Very high HDI countries were responsible for 49.4% of NHL cases and 39.2% of NHL deaths, while medium and low HDI countries accounted for only 17.7% of NHL cases but 22.5% of NHL deaths (**Table 1**). Our study reported more than a doubling of ASIRs in high HDI countries compared with the population in the countries with low HDI. A linear regression depicts the relationship between NHL rates and HDI levels in **Figures 2A, B**. Considering all 185 countries, the ASIRs of NHL rose with HDI levels ( $R^{2}$ = 0.41, P< 0.001) (**Figure 2A**), whereas the ASMRs of NHL were not associated with the levels of HDI ( $R^{2}$ = 0, P = 0.754) (**Figure 2B**).

Significant changes in case fatality percentage can be seen based on the HDI levels, and the incidence-to-mortality ratio increased with rising HDI levels in NHL (**Table 1**; **Supplementary Figure 1**). It was worth noting that the case fatality rates for NHL were higher than 28% regardless of HDI levels, with a corresponding range of 29.0% to 71.1%. By world region, only Australia and New Zealand, North America, and Northern Europe had NHL case fatality rates below 25%. The NHL case fatality rate was significantly higher in low HDI countries, as it varied more than three times from 22.4% in Australia and New Zealand to 72.2% in Middle Africa. Except for Micronesia, Africa spotted the worst-case fatality rate of NHL, which reached an astonishing 67%.

### 3.2 Associations of NHL global incidence and mortality with HIV prevalence and lifestyle risk factors

HIV was generally believed to be a possible contributing factor in the development of certain types of NHL, but the relationship with this broad category of NHL remained unknown. A graphic depiction of HIV prevalence in 2020 and the estimated ASIR of NHL in 2020, both sexes combined, is shown in **Figure 2C**. These estimates of HIV prevalence varied by country and region. **Figure 2C** presents the linear relation between the magnitude of the NHL incidence and the level of HDI, which seems to be restricted to very high HDI countries. A multiple linear regression described the correlation among HDI, HIV prevalence, and ASIR, as offered in **Supplementary Table 3**. According to the beta coefficient (β), both HDI and HIV prevalence strongly correlated with ASIR, while HDI was a more important factor contributing to a higher ASIR.

**Figure 3:**
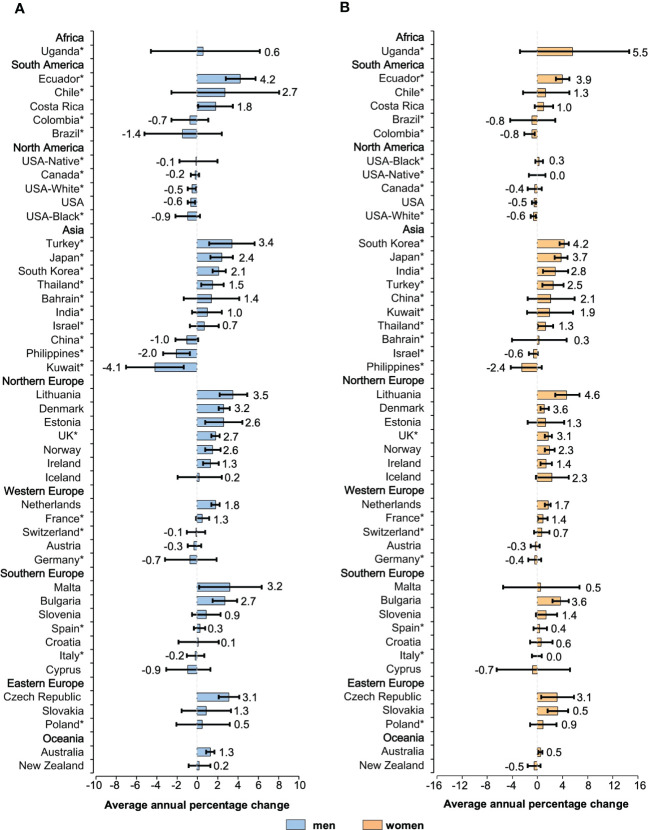
Average annual percentage change for the age-standardized incidence rates of NHL in individuals of all ages. Colored boxes denote the average annual percentage change, and error bars represent 95% CIs. **(A)** men. **(B)** women. *Subnational data. NHL, non-Hodgkin lymphoma; CI, confidence interval.

The increasing prevalence of lifestyle and metabolic risk factors was observed in some countries with high incidence rates, so the correlations between risk factors and NHL development were also estimated (**Figure 3**; **Supplementary Table 4**). According to correlation analyses, only obesity and physical inactivity were strongly associated with ASIR and ASMR of NHL in both genders (**Supplementary Figure 3**). Regarding the multivariate linear regression, among men, higher ASIRs of NHL were associated with a lower prevalence of diabetes (β = -0.707, P<0.001) and hypertension (β = -0.320, P<0.001). Referring to obesity (β = 0.272, P<0.001) and high levels of mean cholesterol (β = 4.506, P<0.001), the increased prevalence of these metabolic factors was accompanied by higher ASIRs (**Supplementary Table 4**). However, smoking and physical inactivity were not statistically meaningful. While among women, a higher incidence of NHL was linked to a higher prevalence of obesity (β = 0.399, P< 0.001), a decreased prevalence of diabetes (β = -0.468, P<0.001), and hypertension (β = -0.386, P<0.001) (**Supplementary Table 4**). However, the analysis did not identify a significant connection between lifestyle behavioral factors, metabolic factors, and ASMR globally (P > 0.05).

### 3.3 Global incidence trends of NHL and comparison by HDI

There was a sign of a slowdown in the increase in NHL incidence in most populations across 42 countries. The initial growth was replaced by a steady trend in countries such as the United States, Canada, and Austria (**Supplementary Figure 2**). Results regarding the incidence of NHL were mixed. It was found that most developed countries had a trend of steady growth, while some low HDI countries had relatively large fluctuations in the incidence. Among men, 30 countries had an increase in incidence (AAPCs, 4.2 to 0.1, **Figure 3A**). Out of all 30 countries with increasing trends, mostly were reported in Europe, with Lithuania (AAPC, 3.5 [95%CI: 2.2 to 4.9]), Malta (AAPC, 3.2 [95%CI: 0.2 to 6.3]), and the Czech Republic (AAPC, 3.1[95%CI: 2.1 to 4.1]) recording the most drastic growth. Considering the countries with a falling trend, the AAPCs of NHL incidence in North America and Oceania were of a lower magnitude. Kuwait (AAPC, -4.1[95%CI: -6.9 to -1.3]), the Philippines (AAPC, -2.0 [95%CI: -3.3 to -0.7]), and Brazil (AAPC, -1.4 [95%CI: -5.1 to 2.4]) showed a downward trend of more than 1%. Among female counterparts (**Figure 3B**), in Uganda, Lithuania, and South Korea, the ASIRs have increased annually by 5.5%, 4.6%, and 4.2%, respectively. These three countries noted the most considerable growth rates in 42 countries. Except for the Philippines (AAPC, -2.4 [95%CI: -4.1 to 0.7]), ASIRs for female NHL decreased on average by less than 1% per year in all populations. In subgroups of different genders, nearly 20 populations exhibited higher ASIRs for women than men.

**Figure 4:**
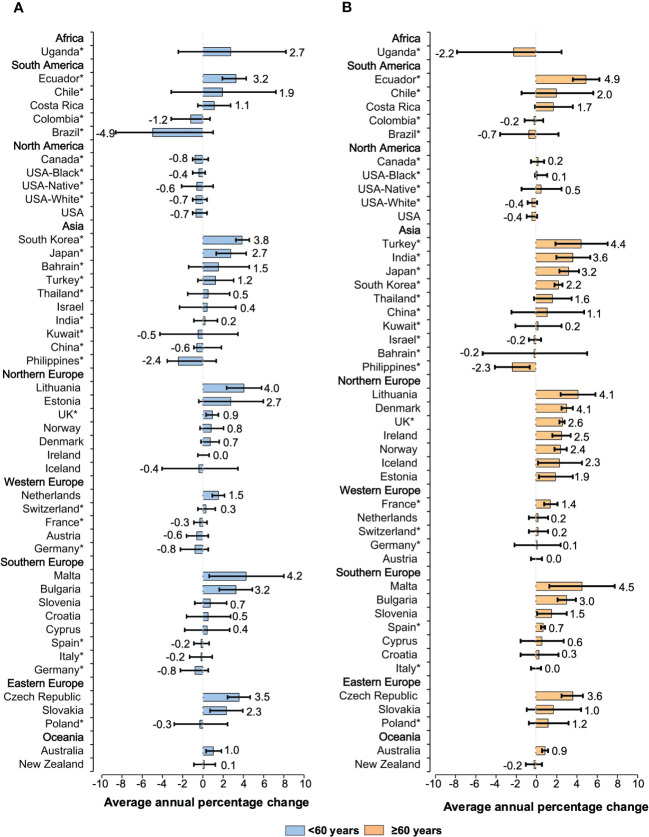
Average annual percentage change for the age-standardized incidence rates of NHL in individuals of both sexes. Colored boxes denote the average annual percentage change, and error bars represent 95% CIs. **(A)** <60 years old. **(B)** ≥60 years old. *Subnational data. NHL, non-Hodgkin lymphoma; CI, confidence interval.

Observed trends also differed across age groups. The incidence of NHL increased in 34 populations (AAPCs, 4.9 to 0.1) among individuals aged 60 years or older (**Figure 4**). The steepest rises in morbidity rates were assessed for Ecuador (AAPC, 4.9 [95%CI: 3.9 to 6.2]), Malta (AAPC, 4.5 [95%CI: 1.4 to 7.7]), and Turkey (AAPC, 4.4 [95%CI: 1.9 to 7.0]). A similar trend has been recorded for individuals younger than 60 years old as ASIRs increased in 27 countries (AAPCs, 4.2 to 0.1). These included Malta (AAPC, 4.2 [95%CI: 0.7 to 7.9]), Lithuania (AAPC, 4.0 [95%CI: 2.3 to 5.7]), and South Korea (AAPC, 3.8 [95%CI: 3.2 to 4.5]). For individuals aged 75 years or older, the rises are pronounced in 33 populations, with the most notable increase seen in Lithuania (AAPC, 6.7 [95%CI: 5.1 to 8.3]), Ecuador (AAPC, 5.6 [95%CI: 2.6 to 8.6]), and India (AAPC, 4.8 [95%CI: 0.5 to 9.3]) (**Supplementary Figure 4**). Approximately half of the countries had AAPCs consistently higher during the 15 years in the subgroup of patients aged 75 years or older.

**Figure 5:**
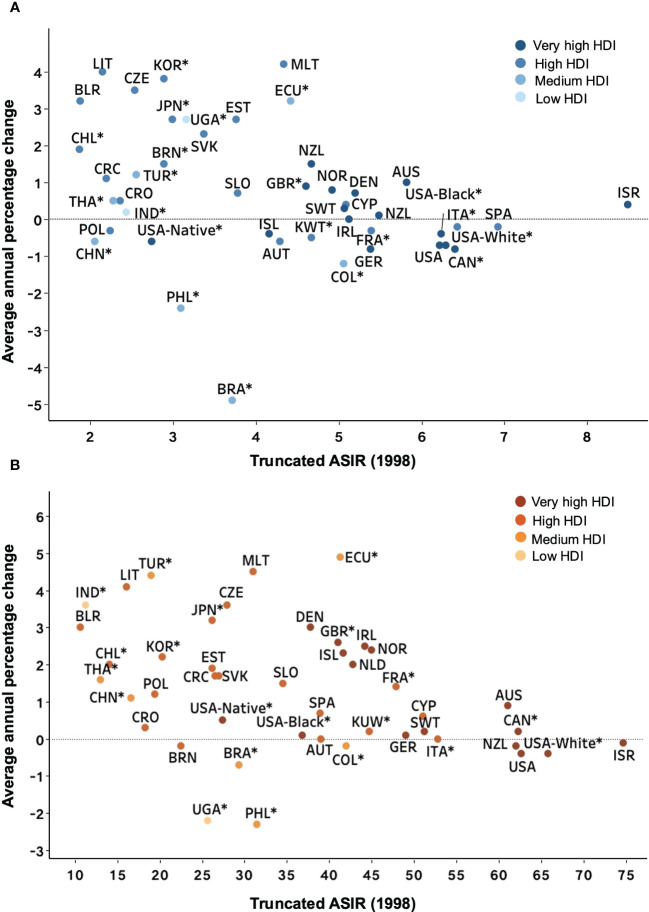
Average annual percentage change for 1998-2012 versus truncated ASIR (world) in 1998 for NHL patients. Country names are represented by the ISO 3166 international standard alpha-3 country code. **(A)** <60 years old. **(B)** ≥60 years old. *Subnational data. ASIR, age-standardized incidence rate; NHL, Non-Hodgkin lymphoma; HDI, Human Development Index.

We noted that while rates stabilized or increased in countries with high ASIRs, the most significant rise was seen in countries with the lowest ASIRs, particularly for people older than 60 (**Figure 5**). However, the relationship between ASIR and HDI was not as explicit, with similar ASIRs presented in low and high HDI countries. Although these countries generally had low HDI, the high AAPCs were noted for Malta, a very high HDI country. As the ASIR in 1998 increased, highly developed countries were clustered around an AAPC of 1% for patients aged less than 60 years old. Conversely, for older patients, a linear decline was recognized in the AAPC as the ASIR in 1998 increased, with the countries with the highest ASIR showing a downward trend over this 15-year study period.

### 3.4 Global mortality trends of NHL

We further analyzed the mortality trends of the NHL over the past decades. Mortality rates have been falling in most countries except for some developing countries, such as Ecuador, where rates have risen over the past years (**Supplementary Figure 5**). Although general declines in mortality rates were seen in those younger than 75 years, the mortality pattern changed significantly in the elderly population. For individuals 75 years or older, Slovakia and South Korea ranked first in Europe and Asia, respectively (**Supplementary Figure 6**). Only several countries from South America and one from Asia (Philippines) reported an increasing AAPC of less than 1%. In contrast to the decreased rates in younger patients in most areas, over half of the populations experienced an increase in the mortality burden of NHL, with notable annual increases ranging from 0.3% to 6.8%. In conclusion, the percentage change in the ASIR varied substantially between countries, but a few countries demonstrated a rise during the measurement period.

### 3.5 Global trends of age-standardized DALY rates and burden attributable to high BMI

**Figure 6:**
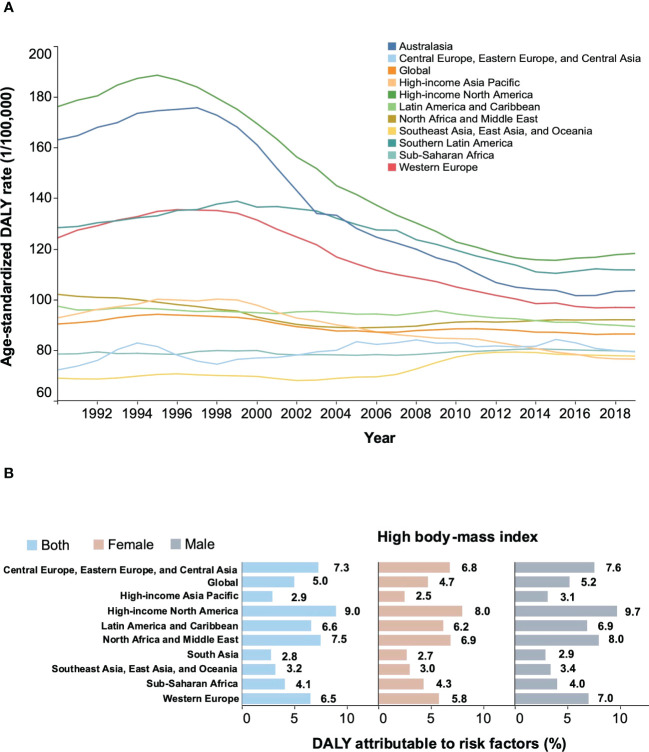
Secular trends of age-standardized DALY rates from 1990 to 2019 and percentage of age-standardized DALYs of NHL attributable to high body-mass index in 2019. **(A)** Secular trends of age-standardized DALY rates for both sexes, 1990-2019. **(B)** percentage of age-standardized DALYs of NHL attributable to high body-mass index in 2019. NHL, non-Hodgkin lymphoma; DALYs, disability-adjusted life years; GBD, Global Burden of Diseases Study.

Overall, NHL contributed 8,650,352 age-standardized DALYs in 2019 worldwide, a decrease of 4.3% compared to 1990 (**Figure 6A**). During this period, age-standardized DALYs have trended downward in some high-income regions, such as North America and the high-income Asia-Pacific. However, over the past ten years, age-standardized DALYs have plateaued globally and in some areas, such as Central Europe and Latin America (**Figure 6A**). Compared with all other regions, Australasia experienced the most significant decline in age-standardized DALY rates between 1990 and 2019, falling by 36.5%. Globally, a high body-mass index was an important contributor to the age-standardized DALYs (7.3%), slightly higher in males than in females (**Figure 6B**). High-income North America has the highest proportion of age-standardized DALYs (9.0%) due to a high body-mass index, which may be three times higher than high-income Asia-Pacific. These findings were consistent with previously identified risk factors associated with morbidity and mortality.

### 3.6 Future global NHL burden in 2040

Globally, the number of new NHL incident cases is projected to increase from approximately 544,000 to an estimated 778,000 by 2040, a corresponding increase of 43% over two decades. Consistent with the findings above, countries or regions with low HDI in transitional economies are more vulnerable, with a striking increase of 84% in incidence from 2020 to 2040 (**Supplementary Figure 7A**). Similarly, low HDI countries are projected to experience a marked increase of 88% in mortality by 2040, according to global rates estimated in 2020(**Supplementary Figure 7B**). Morbidity and mortality projections to 2040 have expanded for the elderly population over 75 years, especially in countries with a high HDI (a 150% increase) (**Supplementary Figure 8**).

## 4 Discussion

There were four main findings from our study. First, the geographical distribution of NHL burden varied substantially, with higher incidence observed in countries with higher HDI. High HDI countries, particularly North America and Australia, account for a sizable portion of NHL’s global morbidity, mortality, and DALYs. Second, a higher incidence was associated with a higher prevalence of HIV infection, obesity, hypertension, and diabetes, whereas mortality was not associated with these risk factors. Third, incidence rates are increasing among females, the elderly, and some populations from Northern Europe, Asia, Africa, and South America. Last, the number of new NHL cases was estimated to increase by nearly 50% over two decades based on demographic projections.

Our findings were generally consistent with incidence and mortality trends in GBD studies, although our estimates were slightly lower than theirs, possibly due to differences in data sources and estimation methods (23). Despite the observed decline in age-standardized DALY rates, this did not necessarily reduce the NHL burden on health systems in high-risk countries. This was because changing age structures and population growth meant that NHL cases and deaths continued to increase in many places (24). We also found that while age-standardized death rates fell substantially in many places, age-standardized incidence rates declined more slowly. For example, age-standardized incidence rates decreased slightly in North America during the study period, while age-standardized mortality and attributable DALY rates experienced relatively significant decreases. The slow decline in incidence may be partially attributed to the continued refinement of diagnostic techniques.

This study found some preventable risk factors associated with the incidence of NHL in both genders, including hypertension, diabetes, and obesity. And these major risk factors are largely preventable. The past few decades have witnessed concurrent increases in the prevalence of obesity and the incidence of NHL, making obesity a suspected risk factor for NHL. A pooled study illustrated that obesity was causally linked to the development of NHL, especially for the DLBCL subtype (25). Indeed, the findings of our analysis, coupled with the evidence from the literature, compellingly showed the causal association between certain lifestyle factors and increased NHL risk. Research has discovered that the westernization of lifestyle may cause the distribution of lymphoma subtypes in Japan to gradually shift to a distribution more like that of the United States, which indicates the impact of lifestyle changes on the incidence pattern (26, 27). The observed increase in the incidence of NHL implied the need for better global cancer prevention in the context of current exacerbated risk factors. Hence, efforts are needed to incorporate effective preventive interventions into existing health plans, as unhealthy lifestyles vigorously promote the development of chronic diseases and cancer.

Notably, another major risk factor for NHL was HIV infection. Lymphomas remained a leading cause of cancer morbidity and mortality for HIV-infected patients, and an elevated risk of developing NHL persists among HIV-infected individuals compared to the general population despite the advent of effective antiretroviral therapy (28). According to registry linkage studies from the USA, Italy, and Australia, the relative risks of NHL in patients with acquired immune deficiency syndrome (AIDS) compared with the general population ranged from 15 for low-grade NHL to 400 for high-grade NHL (29). HIV acts to promote carcinogenesis *via* immunosuppression (8). Specific subtypes of NHL, such as Burkitt lymphoma, have been highly associated with HIV and solid organ transplants (30). The emergence of HIV causes an additional increase in the incidence of NHL. In countries with a high prevalence of HIV, the incidence of NHL was anticipated to increase, and interventions such as closer monitoring and more frequent screening of HIV may be required. AIDS-related lymphomas were associated with poorer outcomes than other lymphomas (31). Although combining chemoimmunotherapy with antiretroviral therapy has considerably prolonged life expectancy in patients with AIDS-related lymphomas, the management of relapsed and refractory disease remained a challenge and an area of unmet need, indicating the necessity of optimization and further development of new treatment strategies (31).

We uncovered that the increase was more evident among women and older populations. One explanation for the rise in female NHL incidence was attributed to a higher prevalence of obesity and a more drastic increase in metabolic syndrome with population aging related to menopause and sex hormones (32). Aging and population growth were other possible drivers of the rapidly increasing burden of the NHL. The shift in demography has led to a significant increase in new cases of NHL. Reductions in DALYs and mortality rates do not necessarily reduce the burden of NHL on the health systems in high-risk regions (24). Given the aging population and the adoption of unhealthy lifestyles, this increasing trend of incidence and mortality rates is projected to continue in many locations. As such, primary prevention through reduced exposure to environmental risk factors, coupled with population-level changes towards healthier lifestyle choices, provides better options for reducing the vast and increasing burden.

There is a mounting demand for highly effective chemotherapy and therapeutic strategies to improve the treatment capabilities of the elderly, as they will have a major social and economic impact on families and society. Given the concurrent comorbidities and the frequent treatment-related complications, older patients are usually treated with attenuated-dose chemotherapy, resulting in a higher risk of treatment failure (33, 34). Thus, novel targeted drugs are emerging to preserve the quality of life and longevity of elderly patients while maintaining effective treatment plans for many years (35). Although new targeted drugs provide options for the treatment of the elderly, it’s still a major clinical issue to improve the efficacy of elderly NHL patients further. Additionally, it was noted that there are plenty of competing risks for clinical events in the elderly, including death due to irrelevant causes (33, 36). A pooled analysis of a population-based cohort depicted the high rates of intensive care unit admission and in‐hospital mortality for aggressive NHL, which may reflect the disease burden on the healthcare system (37). Concerning the decline in the functional status of older patients, caregivers are essential in cancer care management. Taken together, our analysis found that the burden of disease in elderly NHL patients is substantial. Therefore, further improvement in the efficacy of elderly NHL patients is urgently needed.

We noted several limitations in our study. First, the international comparison may be constrained by data sources, as the GLOBOCAN database rarely includes countries with few population-based high-quality cancer registries. Although we used different databases, which might have led to some statistical variations, the overall trend is still consistent. Second, more data collection and precise analyses of morphological subtype, age, ethnicity, and stage at diagnosis of NHL were needed to identify survival differences in large cohorts. As GBD only provided the attribution of a high BMI, other risk factors required further investigation. Third, data quality varied across countries. In many low- and middle-income countries, such as those in sub-Saharan Africa, low-quality data can lead to considerable uncertainties in estimates. Last, projections of the future burden of NHL in 2040 did not involve changes in background incidence over time and histological subtypes and thus only reflected shifts in population growth and aging over the next 20 years.

## 5 Conclusions

In conclusion, the results of our study act as an alert to the growing burden of NHL worldwide, which varies substantially by country. North America and Australia had the highest number of NHL incident cases, followed by Northern Europe in 2020. Although the ASMR of NHL in most countries has declined in recent decades, its burden remains high, especially in developing countries. Considering the aging population worldwide and the lifestyle risk factors people face across their lifespans, the increasing trends will likely continue in the coming decades. Future efforts should be directed toward tailoring cancer control actions following regional risk factor assessments and cancer burden profiles. More detailed investigations into the reasons behind temporal trends and the epidemiology of different subtypes are warranted and could enable policymakers to allocate limited resources and formulate policies more rationally based on these investigations.

## Data availability statement

The original contributions presented in the study are included in the article/**Supplementary Material**. Further inquiries can be directed to the corresponding authors.

## Ethics statement

Ethical review and approval was not required for the study on human participants in accordance with the local legislation and institutional requirements. Written informed consent for participation was not required for this study in accordance with the national legislation and the institutional requirements.

## Author contributions

YC and XZ designed the study. YC, YLiu, XF, YJ, MD, and XG collected the data. YC, DY, KL, and PL analyzed the data. YC drafted the manuscript. YLi, HX, and JF critically reviewed the manuscript. XZ and XW reviewed and revised the manuscript. XW provided direction and guidance throughout the preparation of the manuscript. All authors contributed to the article and approved the submitted version.
