# Supplementary material for: The epidemiological patterns of non-Hodgkin lymphoma: global estimates of disease burden, risk factors, and temporal trends
Source: Front Oncol. 2023 Jun 2;13:1059914. doi: 10.3389/fonc.2023.1059914 (PMC10272809; doi:10.3389/fonc.2023.1059914)
Supplement: Supplementary file 1 [file DataSheet_1.pdf]

The epidemiological landscape of non-Hodgkin lymphoma: a worldwide analysis of incidence, mortality, and risk factors

Supplement information

Contents

Supplementary Table 1. The data source for the prevalence of risk factors 2

Supplementary Table 2. The data source for the trend analysis of NHL.. 3

Supplementary Table 3. The multiple linear regression among HDI, HIV prevalence, and ASIR..........6

Supplementary Table 4. The association with prevalence of lifestyle and metabolic risk factors........... 7

Supplementary Figure 1. Age-specific mortality/incidence ratio of NHL by different countries and comparison by HDI levels 8

Supplementary Figure 2. Trends in age-standardized incidence rates of NHL patients. Cancer truncated ASIRs, 1998-2012, for each country 9

Supplementary Figure 3. The correlation analysis of risk factors for NHL's age-standardized incidence and mortality by sex………... 10

Supplementary Figure 4. Average annual percentage change for the age-standardized incidence rates of NHL in individuals for both sexes.. 11

Supplementary Figure 5. Average annual percentage change for the age-standardized mortality rates of NHL in individuals for different sexes.. 12

Supplementary Figure 6. Average annual percentage change for the age-standardized mortality rates of NHL in individuals for both sexes.. 13

Supplementary Figure 7 Projected number of new cases and deaths for NHL (both sexes combined) in 2040 according to the Human Development Index.. 14

Supplementary Figure 8 Projected number of new cases and deaths for NHL patients aged over 75 years (both sexes combined) in 2040 according to the Human Development Index. 15

Table S1. The data source for the prevalence of risk factors.

Risk factors	Measurement	Year
**Smoking**	Current smoking of any tobacco product (age-standardized rate)	2016
**High cholesterol**	Prevalence of mean total cholesterol (age-standardized rate)	2016
**Physical inactivity**	Prevalence of insufficient physical activity (doing less than 60 min of daily physical activity of moderate-to-vigorous intensity) or as being active for less than 60 min on 5 days per week) (age-standardized rate)	2016
**Obesity**	Prevalence of obesity (BMI>28, age-standardized rate)	2016
**Diabetes**	Raised fasting blood glucose (≥7.0 mmol/L or on medication, age-standardized rate)	2014*
**Hypertension**	Raised blood pressure (SBP ≥140 OR DBP ≥90, age-standardized)	2015*

*Data of this year were selected for these factors as they were not available for 2016. SBP, systolic blood pressure; DBP, diastolic blood pressure.

Table S2. The data source for the trend analysis of NHL.

	Incidence	Mortality
**Australia**	CI5 (1998-2012)	WHO (1998-2018)
**Austria**	CI5 (1998-2012)	WHO (2002-2019)
**Bahrain**	CI5 (1998-2012)	WHO (2002-2011)
**Belgium**	n/a	WHO (1998-2016)
**Brazil**	CI5 (1998-2012)[1]	WHO (1998-2019)
**Bulgaria**	CI5 (1998-2012)	WHO (2005-2018)
**Canada**	CI5 (1998-2012)[2]	WHO (2000-2019)
**Chile**	CI5 (1998-2012)[3]	WHO (1998-2018)
**China**	CI5 (1998-2012)[4]	n/a
**Colombia**	CI5 (1998-2012)[5]	WHO (1998-2017)
**Costa Rica**	CI5 (1998-2012)	WHO (1998-2019)
**Croatia**	CI5 (1998-2012)	WHO (1998-2017)
**Cyprus**	CI5 (1998-2012)	WHO (1998-2017)
**Czech Republic**	CI5 (1998-2012)	WHO (1998-2019)
**Denmark**	CI5 (1998-2012)	WHO (1998-2019)
**Ecuador**	CI5 (1998-2012)[6]	WHO (1998-2017)
**Estonia**	CI5 (1998-2012)	WHO (1998-2016)
**Finland**	n/a	WHO (1999-2018)
**France**	CI5 (1998-2011)[7]	WHO (2000-2014)
**Germany**	CI5 (1998-2012)[8]	WHO (1998-2019)
**Iceland**	CI5 (1998-2012)	WHO (1998-2019)
**India**	CI5 (1998-2012)[9]	n/a
**Ireland**	CI5 (1998-2012)	WHO (1998-2015)
**Israel**	CI5 (1998-2012)	WHO (1998-2018)
**Italy**	CI5 (1998-2010) [10]	WHO (2003-2017)
**Japan**	CI5 (1998-2010)[11]	WHO (1998-2018)
**Korea**	CI5 (1999-2012)[12]	WHO (1998-2019)
**Kuwait**	CI5 (1998-2012)	Missing data
**Lithuania**	CI5 (1998-2012)	WHO (1998-2019)
**Malta**	CI5 (1998-2012)	WHO (1998-2017)
**Netherlands**	CI5 (1989-2012)	WHO (1998-2018)
**New Zealand**	CI5 (1998-2012)	WHO (2000-2016)
**Norway**	CI5 (1998-2012)	n/a
**Philippines**	CI5 (1998-2012)[14]	WHO (1999-2011)
**Poland**	CI5 (1998-2012)[15]	WHO (1998-2018)

**Slovakia**	CI5 (1998-2012)	WHO (1998-2014)
**Slovenia**	CI5 (1998-2012)	WHO (1998-2019)
**Spain**	CI5 (1998-2012)[16]	WHO (2003-2017)
**Sweden**	n/a	WHO (1998-2018)
**Switzerland**	CI5 (1998-2012)[17]	WHO (1998-2017)
**Thailand**	CI5 (1998-2012)[18]	Missing data
**Turkey**	CI5 (1998-2012)[19]	WHO (2009-2016)
**Uganda**	CI5 (1998-2012)[20]	n/a
**United Kingdom**	CI5 (1998-2012)[21]	WHO (2001-2016)
**USA**	CI5 (1998-2012) [22]	WHO (1999-2017) [22]
**USA Black**	CI5 (1998-2012) [22]	n/a
**USA White**	CI5 (1998-2012) [22]	n/a

n/a" not available; CI5: Cancer Incidence in Five Continents V; WHO: World Health Organization.

1. Brazil, Goiania

2. Canada (excl. Nunavut, Quebec and Yukon)

3. Chile, Valdivia

4. China (4 registries)

5. Colombia, Cali

6. Ecuador, Quito

7. France (9 registries)

8. Germany (2 registries)

9. India, Chennai

10. Italy (8 registries)

11. Japan (4 registries)

12. Korea (5 registries)

13. Republic of Korea

14. Philippines, Manila

15. Poland, Kielce

16. Spain (9 registries)

17. Switzerland (6 registries)

18. Thailand (4 registries)

19. Turkey (2 registries)

20. Uganda, Kampala

21. UK, England

22. USA (9 registries)

Table S3 The multiple linear regression among HDI, HIV prevalence, and ASIR.

	**β**	**Standard error**	***P*-value**	**VIF**
**HDI**	3.987	0.855	<0.001	1.032
**Prevalence**	0.097	0.029	0.002	1.032

Abbreviations: HDI, Human Development Index; HIV, human immunodeficiency virus ASIR, age-standardized incidence rate; VIF, variance inflation factor.

ASIR=3.987*HDI+0.097*prevalence+0.406

Table S4 The association with prevalence of lifestyle and metabolic risk factors

Outcome	Risk factors	Male		Female	
		**β**	***P***	**β**	***P***
**Incidence (ASR)**	Smoking	-0.045	0.120	0.010	0.897
	Diabetes	-0.707	**<0.001**	-0.468	**<0.001**
	Hypertension	-0.32	**<0.001**	-0.386	**<0.001**
	High cholesterol	4.506	**<0.001**	0.083	0.383
	Physical inactivity	0.004	0.921	0.132	0.069
	Obesity	0.272	**<0.001**	0.399	**<0.001**
**Mortality (ASR)**	Smoking	-0.021	0.085	-0.132	0.212
	Hypertension	-0.034	0.308	-0.025	0.842
	High cholesterol	0.939	0.075	-0.080	0.566
	Physical inactivity	0.001	0.971	0.188	0.076
	Obesity	0.012	0.564	0.138	0.127

The analysis was conducted using multivariable linear regression model at a country level. The beta coefficient can be interpreted as the change in ASR of incidence or mortality associated with one percent increase of a certain risk factor. Abbreviations: β, beta coefficient; CI, confidence interval; ASR, age-standardized rate.

Figure S1 Age-specific mortality/incidence ratio of NHL by different countries and comparison by HDI levels.

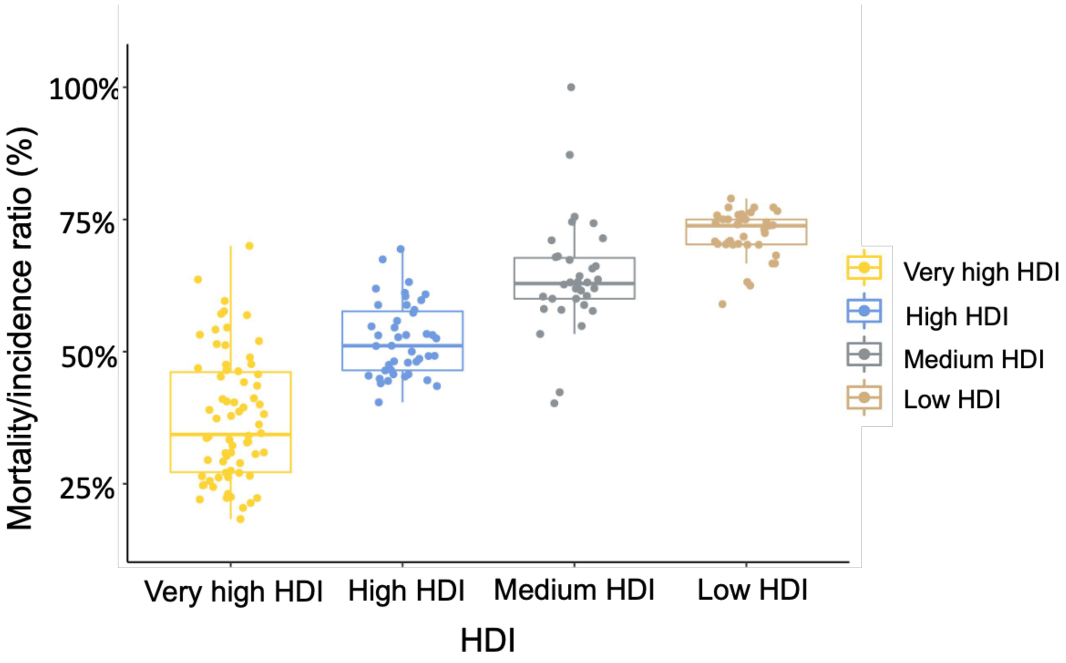

The data was obtained from the GLOBOCAN database in 2020. Abbreviations: NHL, Non-Hodgkin lymphoma; HDI, Human Development Index.

Figure S2 Trends in age-standardized incidence rates of NHL patients. Cancer truncated ASIRs, 1998-2012, for each country.

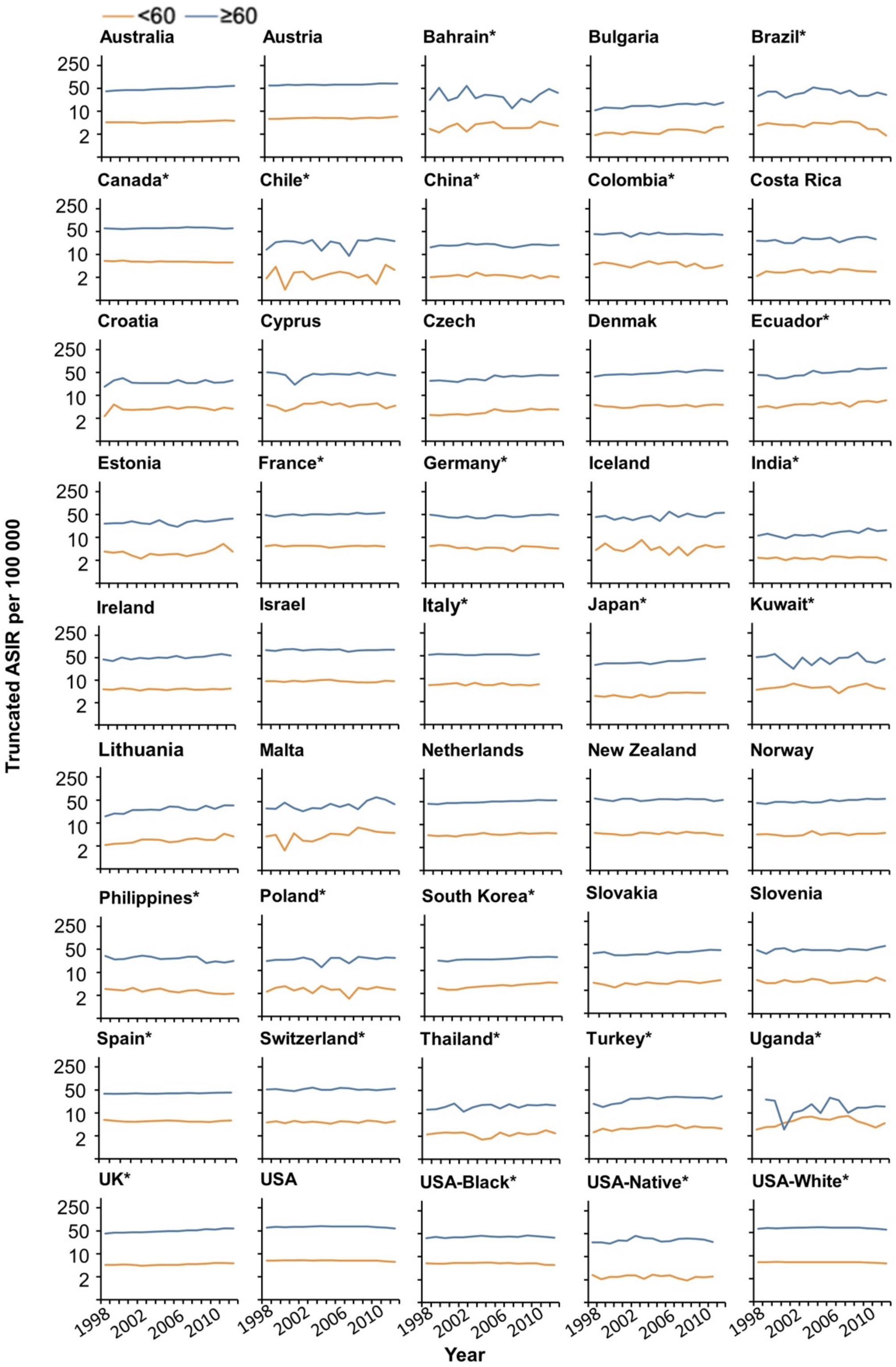

The data was obtained from the CI5plus database and IARC. Abbreviations: NHL, Non-Hodgkin lymphoma; CI5plus, Cancer Incidence in Five Continents Time Trends; IARC, International Agency for Research on Cancer.

Figure S3 The correlation analysis of risk factors for NHL's age-standardized incidence and mortality by sex.

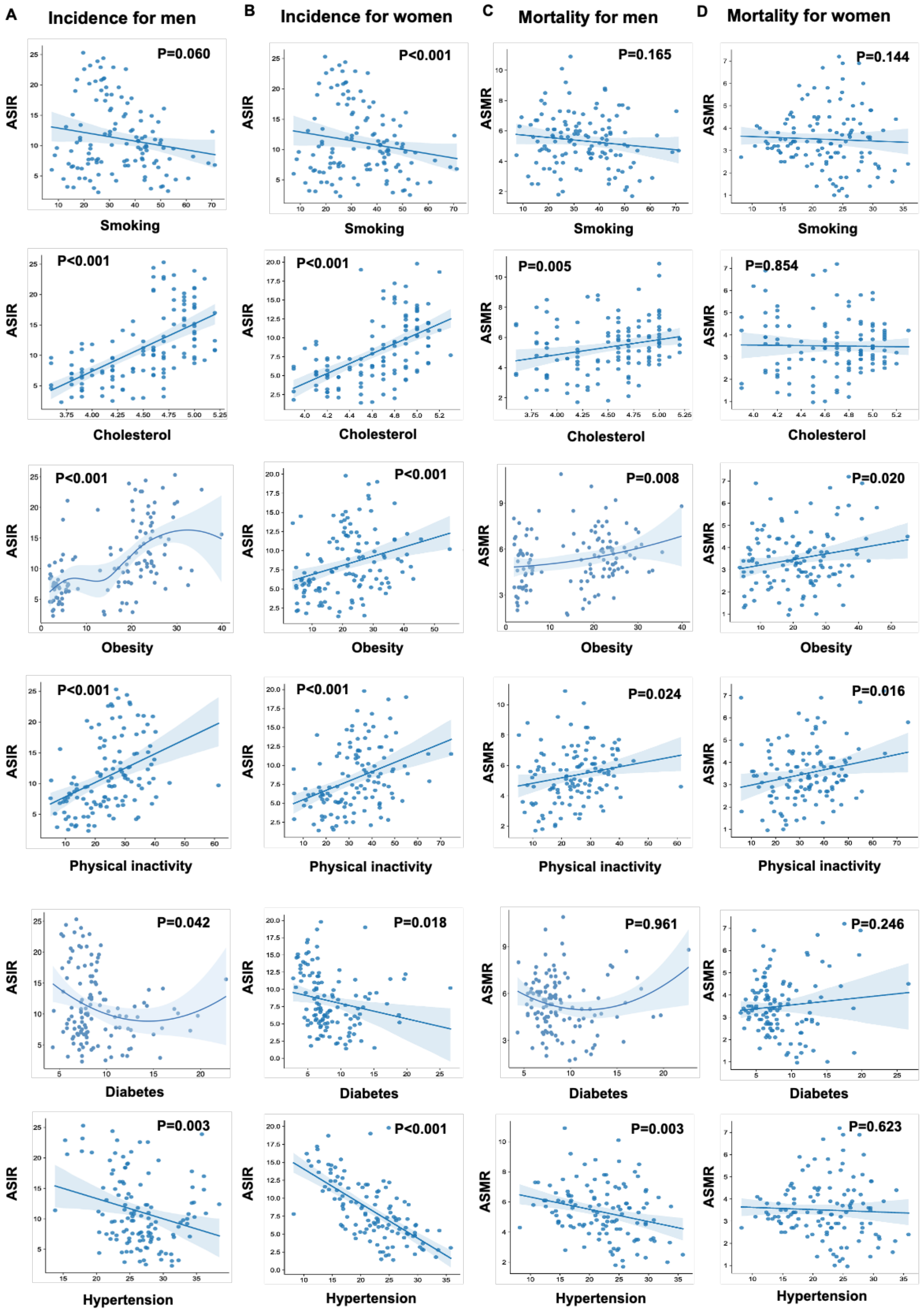

A: Incidence for men. B: Incidence for women. C: Mortality for men. D: Mortality for women. Abbreviations: NHL, non-Hodgkin lymphoma; CI, confidence interval.

Figure S4 Average annual percentage change for the age-standardized incidence rates of NHL in individuals for both sexes.

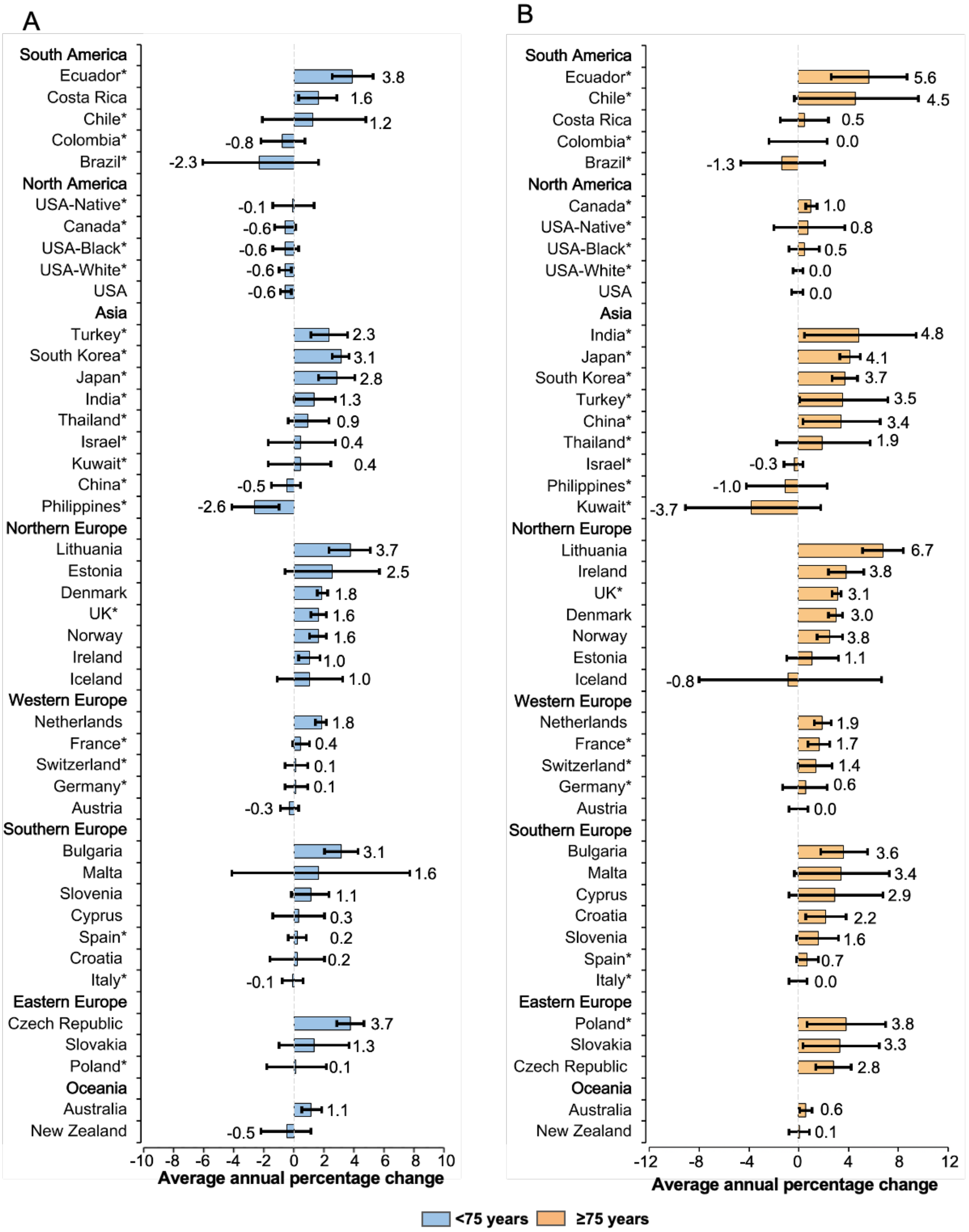

Colored boxes denote the average annual percentage change and error bars represent 95% CIs. A: <75 years old. B: ≥75 years old. *Subnational data. Abbreviations: NHL, Non-Hodgkin lymphoma; CI, confidence interval.

Figure S5 Average annual percentage change for the age-standardized mortality rates of NHL in individuals for both sexes.

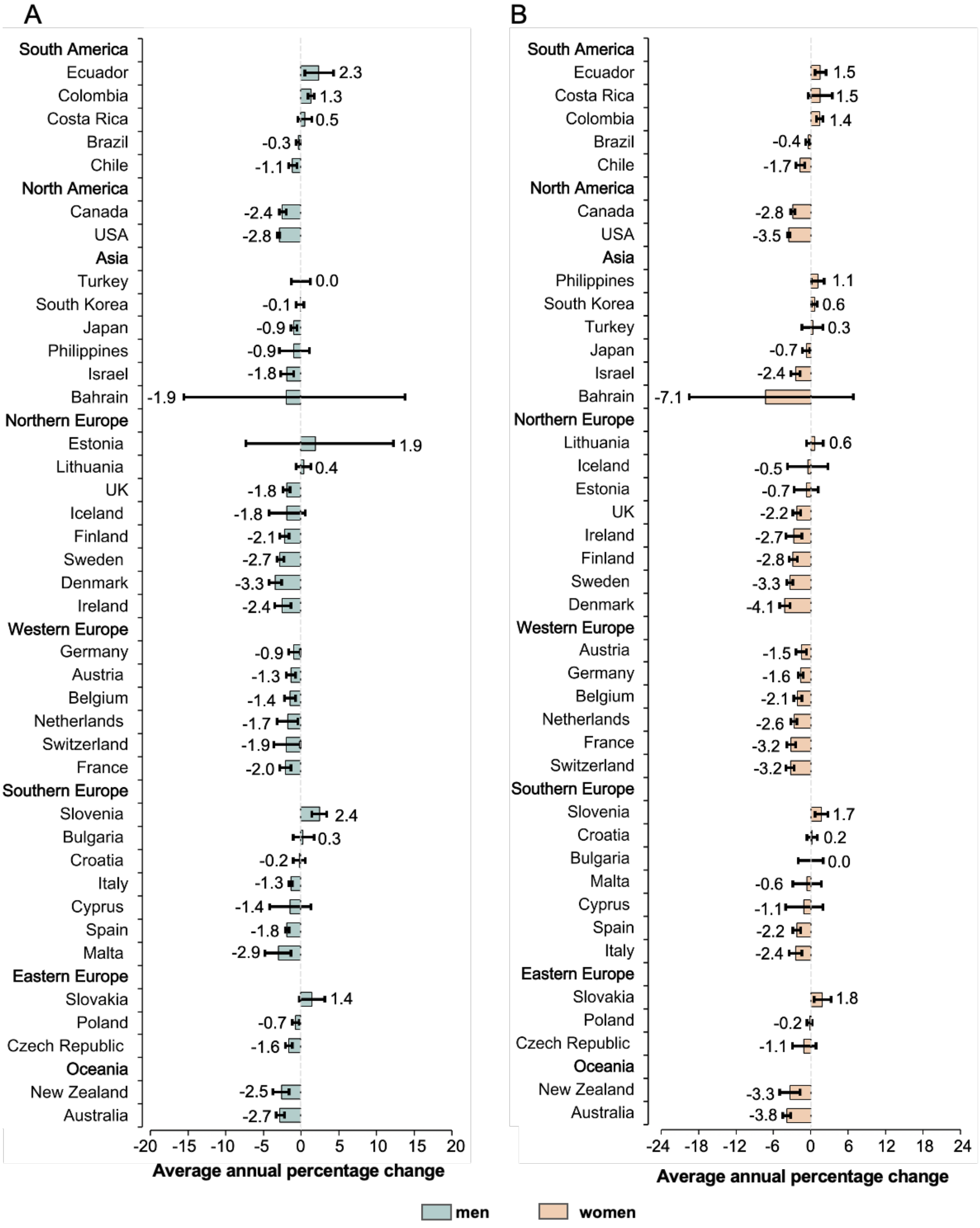

Colored boxes denote the average annual percentage change and error bars represent 95% CIs. A: men. B: women. Abbreviations: NHL, Non-Hodgkin lymphoma; CI, confidence interval.

Figure S6 Average annual percentage change for the age-standardized mortality rates of NHL in individuals for both sexes.

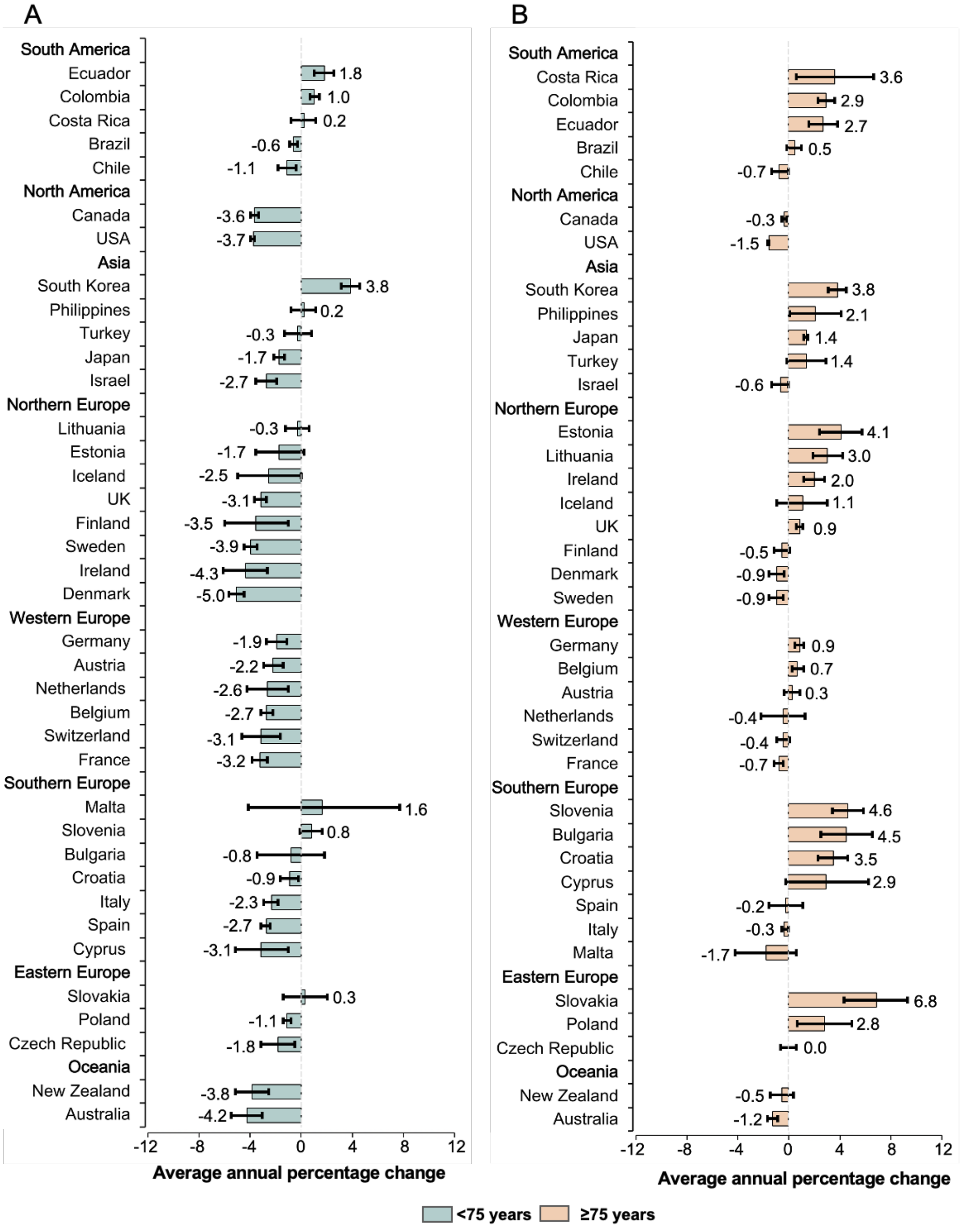

Colored boxes denote the average annual percentage change and error bars represent 95% CIs. A: <75 years old. B: ≥75 years old. Abbreviations: NHL, Non-Hodgkin lymphoma; CI, confidence interval.

Figure S7 Projected number of new cases and deaths for NHL (both sexes combined) in 2040 according to the Human Development Index.

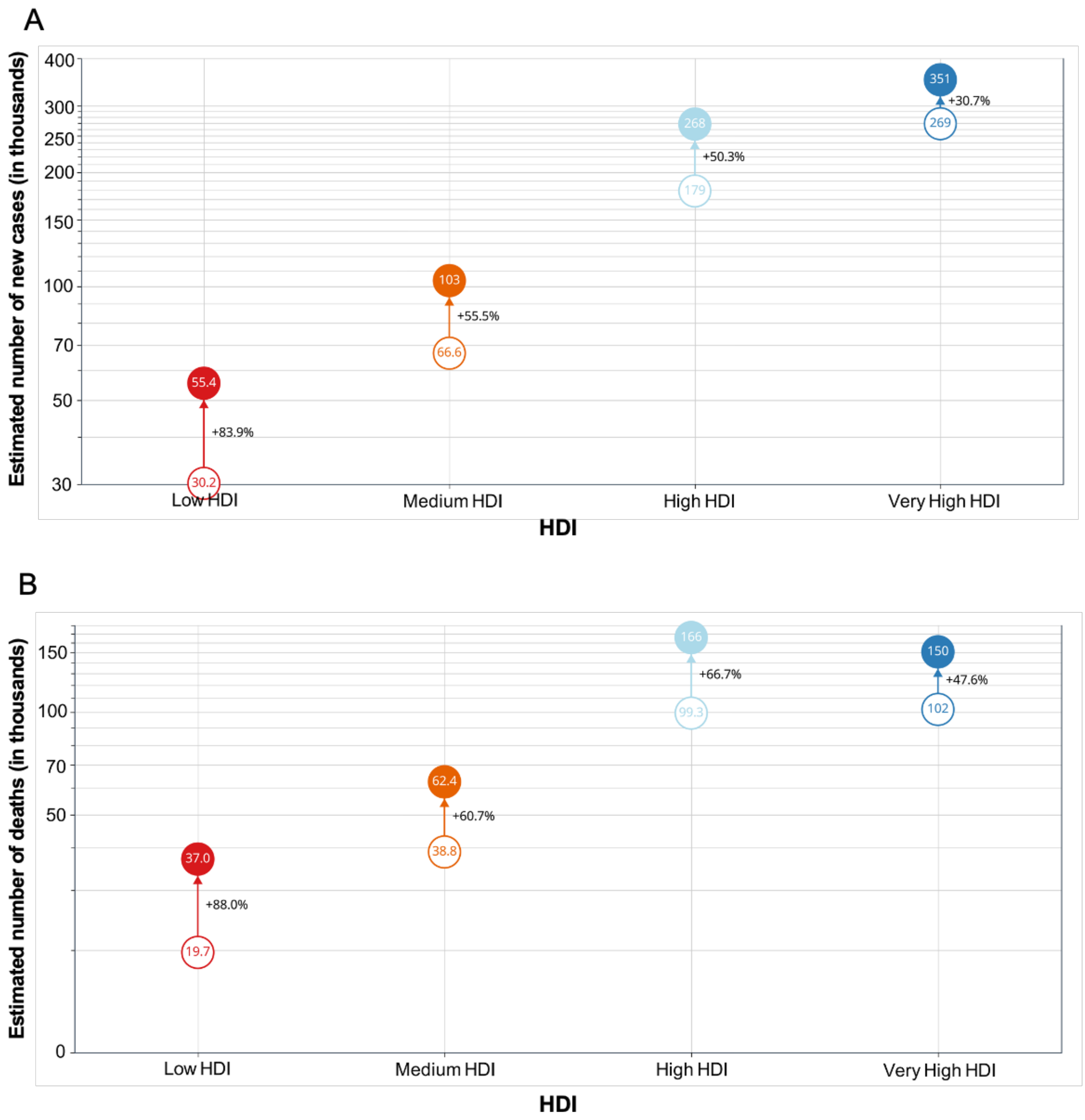

Source: GLOBOCAN 2020. Abbreviations: NHL, non-Hodgkin lymphoma; HDI, Human Development Index.

Figure S8 Projected number of new cases and deaths for NHL patients aged over 75 years (both sexes combined) in 2040 according to the Human Development Index.

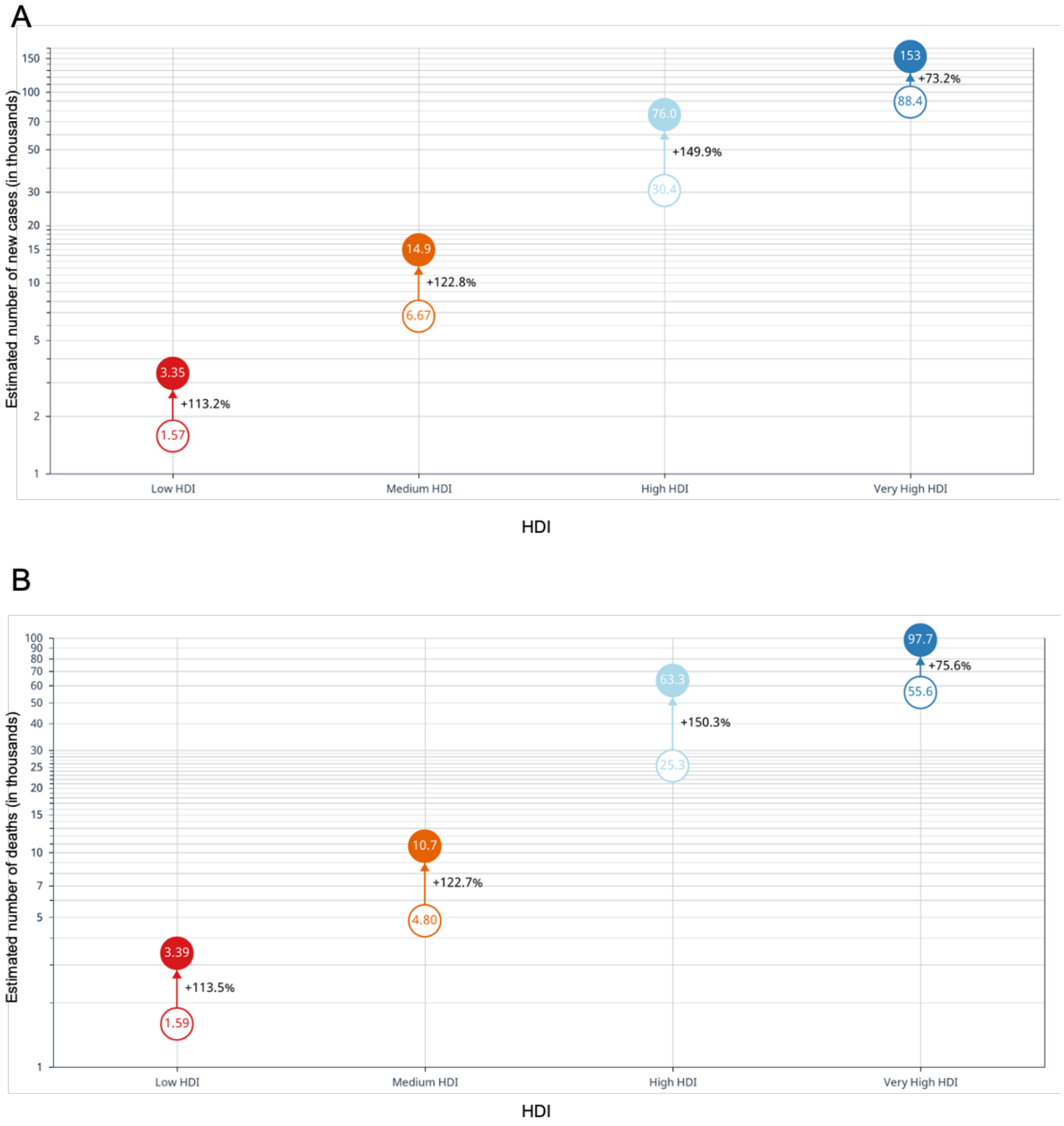

Source: GLOBOCAN 2020. Abbreviations: NHL, Non-Hodgkin lymphoma; HDI, Human Development Index.
